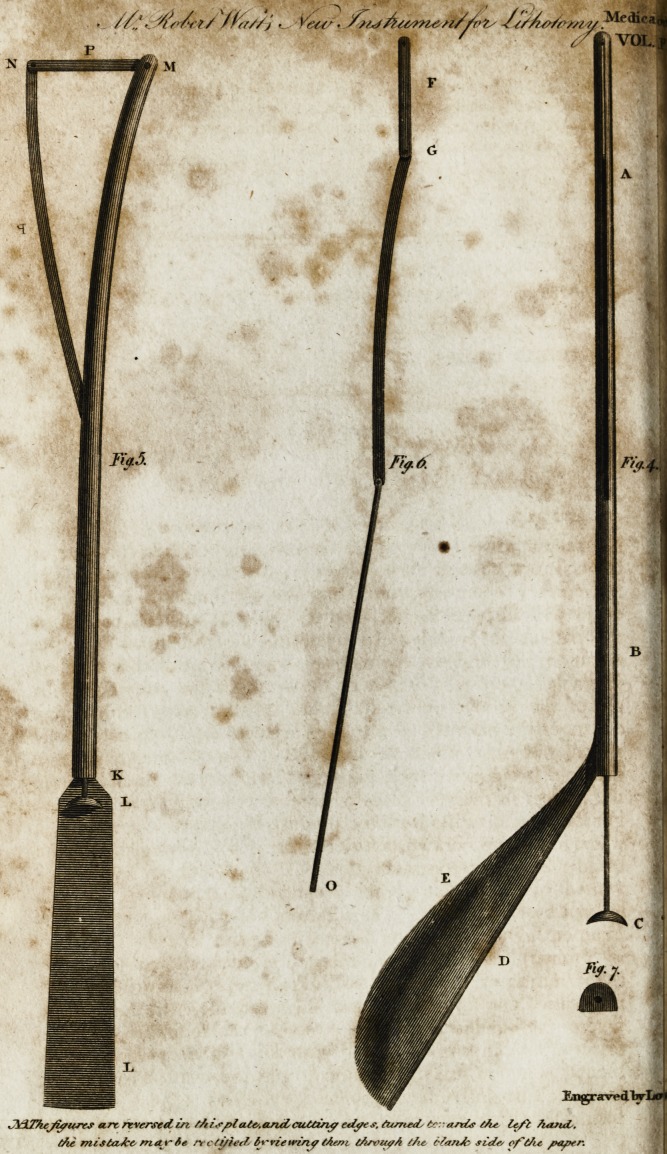# Description of a New Instrument for Operating for the Stone

**Published:** 1800-03

**Authors:** Robert Watt

**Affiliations:** Surgeon in Paisley


### *Deſcription of a new Inſtrument for operating for the Stone. Communicated by* Mr. Robert Watt, Surgeon in Paiſley.

---

### *To the Editors of the Medical and Phyſical Journal.*

Gentlemen,

THE following alteration in the apparatus for Lithotomy has been approved of by ſome eminent ſurgeons in this part of the country, though never as yet tried upon the living ſubject.

In every operation, our chief object ſhould be, to avoid every poſſibility of danger, and to render it as little painful to the patient as poſſible. In operating for the ſtone with the common inſtruments, the danger and pains are both very conſiderable: there is a danger of the beak of the gorget getting out of the groove of the ſtaff, and injuring parts which may endanger the patient's life. This accident has frequently happened, not only to new beginners, but alſo to ſome, who have previouſly operated with ſucceſs. Though we ſhould enter the bladder in the very place where we wiſh, ſtill there is a danger of wounding its back or fundus, if the inſtrument is not immediately withdrawn before the urine be diſcharged, and the bladder begins to contract: this may, in ſome meaſure, be prevented by the double gorget propoſed by Dr. Monro, and improved by Dr. Jeffery. The addition, however, renders the inſtrument bulky, and more difficult to be introduced.

It ſometimes happens too, that the bladder is ſo contracted by the irritation of the ſtone, that it can neither be diſtended by the urine, nor any other means we can uſe. In ſuch caſes, where the fundus has fallen down to the ſphincter, there is ſcarce a poſſibility of operating with any of the common inſtruments, without making an inciſion in the one as well as the other. The inſtrument propoſed by *Frere Coſmé*, would in ſuch caſes, upon entering through the ſphincter of the bladder,

 carry carry the fundus before them in ſuch a manner, that the knife, when raiſed out of the groove, would infallibly make an inciſion in it.

Theſe difficulties ſeem, in ſome meaſure, to be removed by the inſtrument now propoſed, the manner of operating with which is as follows: Having introduced the ſtaff, and made an inciſion upon it, through the membranous part of the urethra, as is done in performing the lateral operation, the inſtrument, with the cutting part concealed, is introduced along the groove of the ſtaff, till it has fairly entered the bladder; the ſtaff may now be withdrawn, and the ſtone being diſtinctly felt by the end of the inſtrument, the thumb is applied to the button, and preſſed forward gradually, raiſing the cutting part out of the groove till it aſſume the form of fig. 5. The edge being now in the proper direction, we withdraw the whole inſtrument, and an opening is made, equal to the diſtance from M. to N. fig. 5, which can be made greater or leſſer, according as we expect a large or a ſmall ſtone, or according to the age of the patient.

With this inſtrument, although the fundus of the bladder ſhould be fallen down to the ſphincter, there will be little danger of its being wounded. For the inſtrument with the cutting part concealed, being introduced two or three inches within the ſphincter, according to the ſize of the bladder, the prop riſing gradually out of the groove till it comes to a right angle with the ſtem, effectually removes any part of the bladder, which may be lying along the ſide of the inſtrument. A circumſtance which renders it greatly ſuperior to that of *Frere Coſmé*. In his, the cutting part goes nearly as far forward as the end of the ſtem, ſo that upon raiſing the knife out of the groove, every thing along the ſide of the inſtrument muſt inevitably be cut through; whereas, in the one now propoſed, the cutting part, when it begins to riſe out of the groove, is at leaſt an inch, or an inch and a quarter, behind the end of the ſtem.

Another and very important advantage attending this inſtrument is, that it cuts eaſier than the cutting director, or common gorget. It often happens, that a conſiderable degree of force muſt be applied, in order to run the gorget forward into the bladder; and owing to its elaſticity, it flies before the edge of the inſtrument, ſo that a fair inciſion can ſcarcely be made: whereas, by evolving the inſtrument within the bladder, and drawing it out, an inciſion can be made with the greateſt eaſe. Having once felt the ſtone with the point of the inſtrument, and elevated the blade, all we have to do, is to cut what is between it and the outſide, ſo that a miſtake can ſcarcely happen. Here it is impoſſible for the bladder, as in the other caſe, to to recede by its elaſticity from the edge of the inſtrument, or the inſtrument to miſs the bladder, or wound it in a wrong place. In this part of the operation with the common gorget, we are working very much in the dark. From our knowledge of the anatomy of the parts, we flatter ourſelves, that it is impoſſible we can go wrong; but alſo, the fatal blunders which are daily committed, ſhow us how far we are miſtaken.

In ſome caſes, after the inciſion is made with the gorget into the bladder, the ſtone being conſiderably larger than we expected, hence we find it impoſſible to contract it, without either lacerating the parts, or enlarging the wound by a ſecond inciſion; the former ought undoubtedly to be avoided, and the latter can ſcarcely be accompliſhed with ſafety, by any of the common inſtruments. For, after an effort has been made to extract the ſtone, the bladder contracts ſo cloſely, that the inciſion cannot be enlarged, either with a ſcalpel or gorget, without cutting through both its ſides. This, however, may be done with the inſtrument now propoſed, by introducing one of a larger ſize, with the cutting part concealed two or three inches within the ſphincter, as at firſt; then reaching forward the fore finger of the left hand to guard the edge, till it reach the external angle of the firſt inciſion; then withdrawing the whole, the wound will be enlarged equal to the difference between M. N. in the two inſtruments.

I may obſerve, that if this inſtrument, upon further trial, ſhall be found to anſwer the purpoſe as well upon the living ſubject as it has been found to do upon the dead, it will render the operation ſafe, and the apparatus extremely ſimple. In operating upon the female, nothing further will be neceſſary than a pair of forceps to extract the ſtone. Upon the male, a ſtaff, a ſcalpel, and a pair of forceps, will form the whole apparatus. Nothing enhances the value of an inſtrument ſo much as the ſimplicity of its conſtruction, and the eaſe and ſafety with which it can be uſed. Theſe were the objects I had in view; how far I have ſucceeded, experience only can determine. I am,

GENTLEMEN,

Your moſt obedient humble ſervant,

*Paiſley, Dec.* 29, 1799. R. WATT.

### EXPLANATION OF THE DRAWING.

Fig. IV. repreſents the inſtrument of a middle ſize, with the prop and cutting part concealed in the groove A. D, a plate of ſteel continued from the end of the ſtem B, covering the wooden part of the handle, E. The handle would perhaps anſwer the purpoſe much better, were it a little longer than it is here repreſented. Figure II. gives a front view, having the button button C pressed forward to K, by which the blade F, and prop P, are elevated out of the groove, and in the position in which they should be, when the operator withdraws the instrument from the bladder. M, a screw, which being taken out, and the button C screwed off the prop and blade, may be withdrawn from the groove and cleaned. Fig. VI. represents the prop, cutting part and wire, taken out from the instrument. By making the joint G with a small screw, and having two or three props of different lengths, the same instrument will then serve to make a large or a small incision, as the case may require. Fig. VII. the button screwed off from the end of the wire O. This ought to be made hollow upon the outside, with rough edges, so that the thumb of the operators may not readily slip off.

I have sometimes thought, that a small spiral spring within the instrument at B, might be of service. It would slightly resist the pressure of the thumb, when applied to the button, by which means the cutting part could be more steadily elevated; and when the thumb was removed, it would replace the whole in their former position. Owing, however, to the smallness of the tube at B, the maker would perhaps find it difficult to give it this addition.

## Figures and Tables

**Fig.5. Fig.6. Fig.4. Fig.7. f1:**